# Conducting Polymer Grafting: Recent and Key Developments

**DOI:** 10.3390/polym12030709

**Published:** 2020-03-23

**Authors:** Nabasmita Maity, Arnab Dawn

**Affiliations:** 1 Institute of Chemistry, The Hebrew University of Jerusalem, Jerusalem 91904, Israel; nabasmitamaity.89@gmail.com; 2 James Winkle College of Pharmacy, University of Cincinnati, Cincinnati, OH 45267-514, USA

**Keywords:** conducting polymer, graft-copolymer, synthesis, supramolecular, post-modification

## Abstract

Since the discovery of conductive polyacetylene, conductive electroactive polymers are at the focal point of technology generation and biocommunication materials. The reasons why this research never stops growing, are twofold: first, the demands from the advanced technology towards more sophistication, precision, durability, processability and cost-effectiveness; and second, the shaping of conducting polymer research in accordance with the above demand. One of the major challenges in conducting polymer research is addressing the processability issue without sacrificing the electroactive properties. Therefore, new synthetic designs and use of post-modification techniques become crucial than ever. This quest is not only advancing the field but also giving birth of new hybrid materials integrating merits of multiple functional motifs. The present review article is an attempt to discuss the recent progress in conducting polymer grafting, which is not entirely new, but relatively lesser developed area for this class of polymers to fine-tune their physicochemical properties. Apart from conventional covalent grafting techniques, non-covalent approach, which is relatively new but has worth creation potential, will also be discussed. The aim is to bring together novel molecular designs and strategies to stimulate the existing conducting polymer synthesis methodologies in order to enrich its fascinating chemistry dedicated toward real-life applications.

## 1. Introduction

**Scheme 1:**
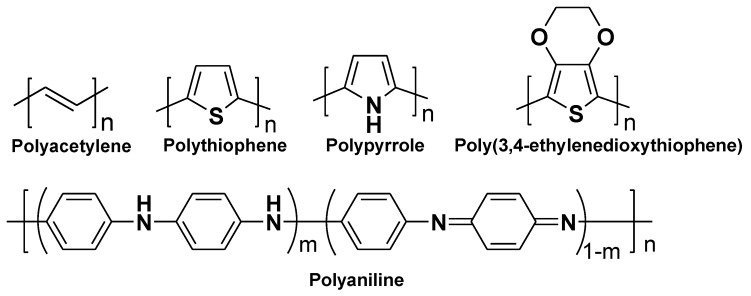
Chemical formulae of most widely used conducting polymers.

Conducting polymers are considered as the fourth generation of polymeric materials. The physicochemical properties inherited from typical polymeric materials combined with the fascinating electrical conductivity approaching metals in cases, brought this class of materials in the focal point of scientific research towards energy applications for decades [1,2,3,4,5,6]. Apart from energy application centered on electrical and electronic properties, conducting polymers are becoming increasingly attractive towards various biomedical applications too due to their responsive chemistry in presence of electrical fields from various types of tissues, including muscle, connective tissue, epithelium and nervous tissue [7,8,9,10,11]. The opportunity to synthesize new conducting polymers with improved and desired properties began to attract the attention of synthetic chemists in the 1980s. Since then a wide range of conducting polymers and their derivatives are synthesized. Some of the most widely used and popular conducting polymers are polyacetylene, polythiophene, poly[3–ethylenedioxy)thiophene], polypyrrole, and polyaniline (Scheme 1).

A key and unique property of a conducting polymer is the presence of conjugated double bonds along its backbone. However, conjugation alone is not enough to make a polymer conductive. The other prerequisite is that some charge carriers in the form of extra electrons or holes have to be injected into the material. Such doping not only induces carriers into the electronic structure but also leads carrier delocalization along the polymer chain and to charge carrier mobility, which is extended into three dimensions through interchain electron transfer [12]. During the doping process, an organic polymer, either an insulator or semiconductor having a small conductivity typically in the range 10^−10^ to 10^−5^ S cm^−1^, is converted into a polymer which is in the ‘metallic’ conducting regime (ca. 1 to 10^4^ S cm^−1^).

The physicochemical properties of a conducting polymer crucially depend on several factors such as: the chemical identity of the monomer, molecular weight, presence of functional groups and side chains attached to the monomeric units, and even on the polymer conformation. On the other hand, the conduction property of the polymer is directly influenced by any factors affecting the electron delocalization along the main chain. Therefore, designing a conducting polymer is much more than only overcoming the synthetic complexity. Time to time functionalization of conducting polymers is perhaps more challenging compared to the synthesis of the parent molecules [7,13]. There are various available routes for conducting polymer synthesis such as chemical [14,15,16,17], electrochemical [18,19,20,21], photochemical [22,23], metathesis [24,25], emulsion [26,27,28], plasma [29,30] etc. While synthesis of the polymer is the important first step, frequently the resultant polymer lacks in desired properties at the desired levels. Processability, morphology, stability, durability etc. are some of the issues, which are extremely vital in the field of technology generation based on the conducting polymer. Such properties are not always solely dependent on the chemical nature of the polymer backbone but on other factors such as availability of the functional groups, nature of the side chain etc. Therefore, engineering chemical structure by keeping the polymeric backbone intact is a well-established strategy for fine-tuning the conducting polymer physicochemical properties. The side chains in conjugated polymers are primarily beneficial as solubilizing groups. However, the overall contribution of side chains is truly far-reaching and a side chain modification can directly influence the optical, electronic, conformational and electrical properties [31]. Therefore, sidechain functionalization of conducting polymers is a popular approach to fine-tune the polymer property. While the polarity (electron withdrawing vs. electron donating) of the sidechain can influence the main chain charge transport, the steric factor of the functional group can alter the planarity and conformation of the main chain thereby influencing the conjugation behavior. On downside amplification of a particular property by side chain modification frequently sacrifices the conduction efficiency of the parent polymer.

The physicochemical property of a polymer can be engineered even more drastically by making a copolymer with another polymer or with the help of grafting. Conducting polymers as the components in block-copolymer systems have been reported for nanostructural control as alternative to lithography [32]. However, the conducting property can severely be scarified in this approach because of dilution effect from nonconducting components. Grafting of the polymers [33,34] on the other hand is an old and classical approach; however, this strategy with conducting polymer is still new and under development. Grafting of conducting polymer is particularly important, as it does not alter the extended conjugated structure in the main chain, however, is capable of introducing and integrating the properties from the grafted materials. Grafting can potentially compensate and improve the properties of the conducting polymers beyond its charge transport, and therefore it could be more associated with solubility, nanodimensional morphology, biocompatibility, biocommunication etc. Especially, in order to qualify for technological or biomedical application conducting polymers need several other factors than only electron delocalization. In this question, it is hard to find the alternatives of grafting with other conventional nonconducting polymers frequently visible in industrial and biomedical sectors.

This review specifically aims to bring together the novel strategies built around conducting polymer grafting to stimulate the real-life applications of this class of materials. We will emphasize on key and recent report based on novelty in strategy, urgency in terms of application and variations in grafted materials. While covalent grafting is a more conventional approach noncovalent immobilization of polymeric counterpart with the parent polymer is no longer a fantasy either. Therefore, we will also discuss the importance and the essence of noncovalent grafting of conducting polymer with the help of limited examples. Ultimately, our goal will be to critically assess and demonstrate at the same time, how the power of grafting can revolutionize the fate of conducting polymer chemistry from academic interest to technology generation and biocommunication.

## 2. Covalent Grafting of Conducting Polymers

**Figure 1:**
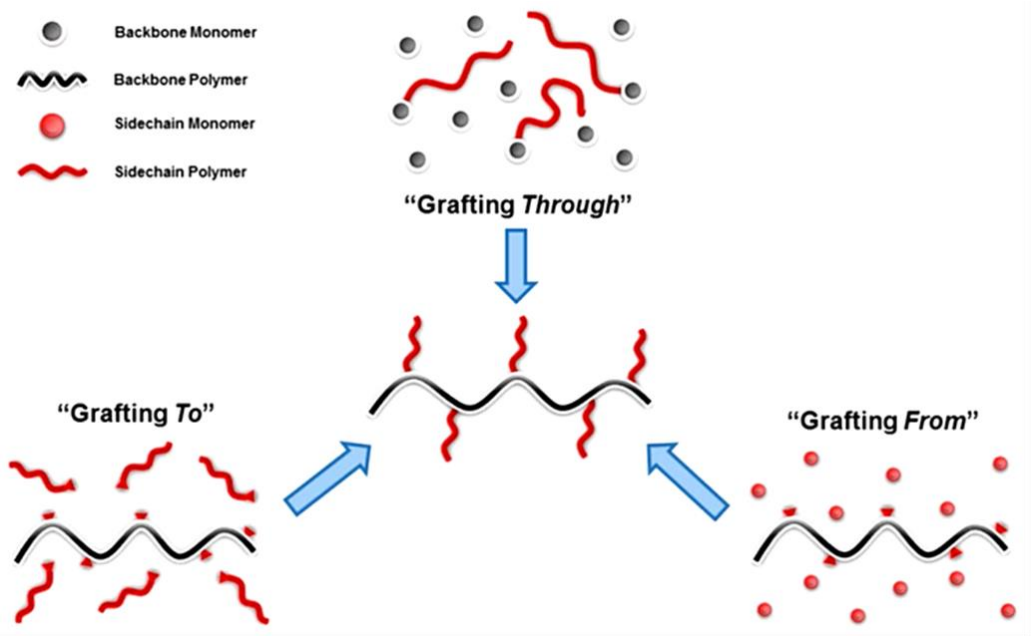
Generalized categories of graft copolymer synthesis: grafting through (macromonomer approach), grafting from (macroinitiator approach), and grafting to. Reproduced with permission [35].

The covalent approach for the synthesis of graft copolymers is the conventional method of grafting. It ensures a highest degree of property mixing among associated species and thus, offers the scope of material design as per the requirement. General methods to synthesize graft copolymers are of three types: (i) ‘grafting to’, (ii) ‘grafting from’ and (iii) ‘grafting through’ (Figure 1) [35]. In brief, ‘grafting to’ method involves attachment of pre-polymerized chains to backbone polymer having reactive end-groups [36]. In ‘grafting from’, conducting polymer backbone functionalized with initiation sides acts as macroinitiator, from which the side chains are grown afterwards [37]. ‘Grafting through’ method involves synthesis of macromonomers that form backbone polymer after subsequent polymerization [38].

**Scheme 2:**
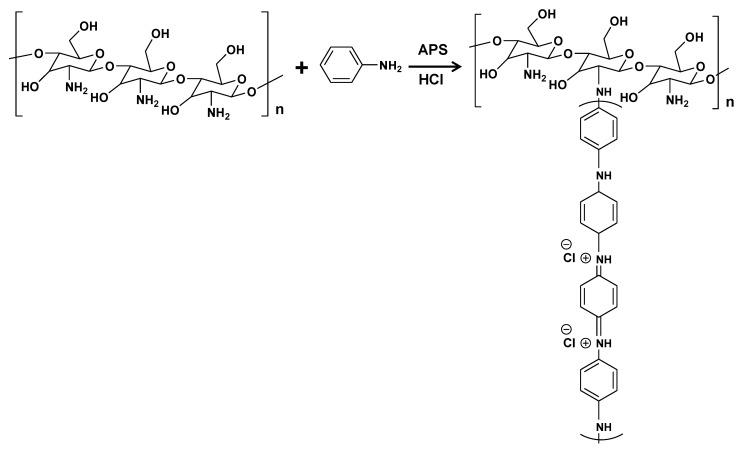
Grafting of polyaniline on chitosan.

Selected works on covalent grafting of conducting polymers are summarized in Table 1. Among various polymerization techniques available in the literature, the most frequently used methods for the synthesis of conducting polymers are oxidative-radical coupling in presence of oxidant catalyst [39] and electrochemical polymerization at electrode surface [40]. For example, Khairkar et al. [41] prepared chitosan–*graft*–polyaniline via oxidative polymerization of aniline in acidic medium using ammonium persulfate (APS) catalyst (Scheme 2). This approach resulted in a cost-effective, environmentally benign conductive biomaterial for sensing application. In a similar way, Abd El-Salam et al. grafted poly(2-hydroxyaniline) and poly(2-methylaniline) on chitosan for the wastewater treatment [42,43]. Polyacrylamide–*graft*–poly(2-methoxyaniline) [44] was also developed by the same group to adsorb lead selectively from contaminated water. Pandey et al. [45] modified natural polymer xanthan gum (XG) by grafting polyaniline (PANI) on it. This XG–*g*–PANI was reported to show high response and recovery times (in order of 10–30 s) in ammonia vapor (concentration range: 1–100 ppb), even at room temperature. Thus, it can be used as a promising ammonia sensor.

**Scheme 3:**
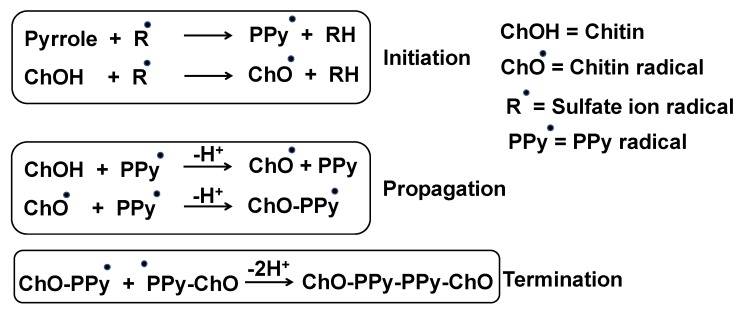
Proposed mechanism of grafting of PPy onto chitin.

Moreover, Ramaprasad et al. [46] followed similar approach in order to prepare more processable polypyrrole (PPy) by grafting PPy onto chitin (Scheme 3) using APS. The authors verified the grafting of PPy by dissolution studies of as prepared material and calculated the % grafting of PPy on chitin via gravimetric method. In another example, Rezaei et al. [47] prepared novolacs grafted with PANI to develop a new adhesive. For the synthesis, they performed grafting of novolacs with p-aminobenzoic acid to introduce amine groups to the novolacs, from which further polymerization of aniline was initiated to form the final graft polymer. After grafting, the authors found a decrease in tensile strength and elasticity. However, the grafting appeared to have no effect on the conductivity of PANI, making the graft polymer an interesting candidate as conductive adhesive. In addition, Smirnov et al. [48] prepared polyacrylamide–*g*–polyaniline electroconductive fibrous mat by electrospinning of copolymers in a water-dimethyl formamide mixed solvent. This fibrous mat can be utilized as a potential electrode material for supercapacitors.

**Figure 2:**
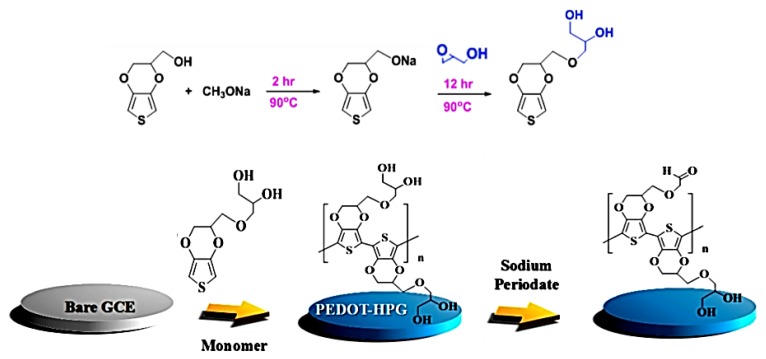
Synthesis route of the new EDOT-based monomer (top), and electrochemical synthesis of PEDOT–*graft*–HPG. Reproduced with permission [49].

**Figure 3:**
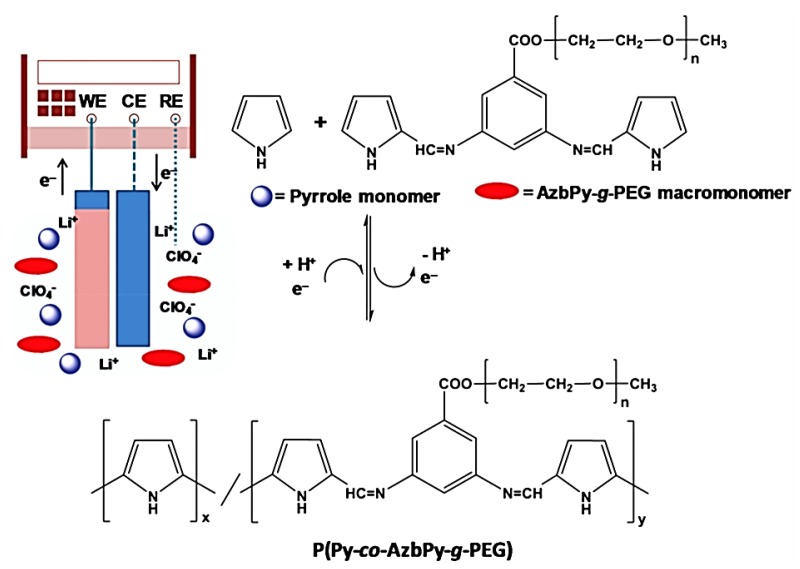
Scheme illustrating the anodic polymerization of P(Py–*co*–AzbPy–*g*–PEG). Reproduced with permission [50].

Using the electrochemical polymerization approach, conducting polymer poly (3,4-ethylenedioxythiophene) (PEDOT) grafted hyperbranched polyglycerol (HPG) was synthesized by Ma et al. [49]. To synthesize the graft conducting polymer (Figure 2), the author introduced hydroxyl groups containing side chains to develop 3,4-ethylenedioxythiophene (EDOT) based monomer, which after subsequent electropolymerization on glass carbon electrode followed by the attachment of alpha-fetoprotein (AFP) antibodies produced antifouling and conducting biosensors. Very recently, Molina et al. [50] designed and developed an amphiphilic conducting graft copolymer having “rod-coil” type randomly distributed conducting backbone of PPy, poly(Schiff base) (PSB) and hydrophilic poly(ethylene glycol) (PEG) side chains. For the synthesis, they prepared bis (pyrrole) benzoic Schiff base-containing PEG macromonomer (AzbPy–*g*–PEG), which after subsequent electrochemical co-polymerization with pyrrole monomers produced the desired polymer P(Py–*co*–AzbPy–*g*–PEG) (Figure 3) capable of generating implantable electrodes for serotonin detection. This macromonomer approach was also employed by Hatamzadeh et al. [51] for the grafting of PPy on thiophene-functionalised polystyrene (PS) macromonomer via the oxidative polymerization of pyrrole monomer. The graft conducting polymer is more solution processable compared to PPy itself. Also, grafting with non-conjugated polystyrene was reported not to affect the characteristic redox behavior of PPy.

**Figure 4:**
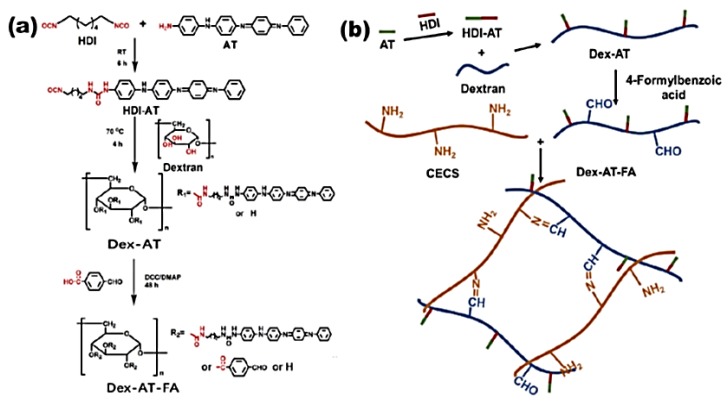
(**a**) Synthesis of dextran–*graft*–(aniline tetramer)–*graft*–(4-formylbenzoic acid), (**b**) Preparation of conducting self-healing hydrogel. Reproduced with permission [52].

In another approach, Guo et al. [52] took tetramer of aniline (AT) instead of PANI to prepare dextran–*graft*–(aniline tetramer)–*graft*–(4-formylbenzoic acid). First, they synthesized hexamethylene diisocyanate-graft-AT and then substituted with dextran. In the next step, 4-formylbenzoic acid was grafted (Figure 4). With this copolymer they developed degradable, conductive, self-healing injectable hydrogel in N-carboxyethyl chitosan solution for myoblast cell therapy and muscle repair. The same research group offered another hydrogel consisting of *N*-carboxyethyl (oxidized hyaluronic acid)–*graft*–(PANI tetramer) [53] for the delivery of antibiotic (amoxicillin). The hydrogel was reported to possess good antibacterial, wound healing properties and it can also prevent wound infections. The gels with higher AT contents showed faster wound healing processes compared to the one with less amount of AT.

**Figure 5:**
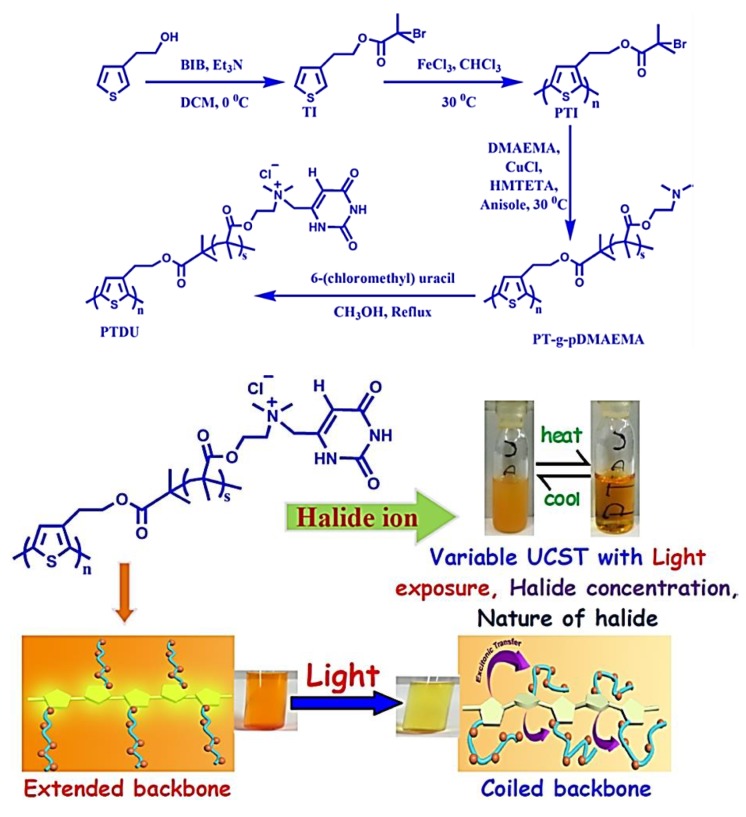
Synthesis of PTDU from 3-(2-Hydroxyethyl)thiophene. Reproduced with permission from [58].

Controlled radical polymerization such as Atom transfer radical polymerization (ATRP), reversible addition-fragmentation chain transfer polymerization (RAFT) is another popular and well accepted technique to develop a variety of conducting polymers-based brushes for wide range of applications. Grafting of side chains not only increases the solubility and processability of conducting polymers but also introduces modulation of properties by external stimuli such as temperature, pH, salt concentration, and electrical potential etc. Conducting polymer grafting can also be done by click reactions [54,55]. Using ATRP technique, Malmstrom et al. [37] successfully grafted pH responsive poly(acrylic acid) brushes from PEDOT backbone. To generate ATRP initiating site, the monomer of PEDOT was functionalized with 2-bromopropanoate. This monomer then was electropolymerized to get bromo-functionalized PEDOT, which undergoes ATRP of tert-butyl acrylate (tBA). PAA brush was obtained after acid hydrolysis of tert-butyl group. The conducting brush was reported to exhibit pH sensitivity, improved aqueous solubility and redox behavior at basic pH, thus can be utilized as functional biointerface and versatile cell culture substrate. Moreover, Zhao et al. [56] reported grafting of neutral poly((oligo(ethylene glycol) methacrylate), poly(OEGMA), and zwitterionic poly([2-(methacryloyloxy)ethyl]dimethyl-(3-sulfopropyl)ammonium hydroxide), poly(SBMA) from bromo-containing PEDOT. A general electropolymerization was done prior to grafting reaction to attach initiating -Br sites from PEDOT and then polymer brushes were grafted via surface-initiated ATRP(SI-ATRP). These brushes were reported to prevent cell attachment and adhesion on the copolymer grafted surface. A self-templating SI-ATRP technique in combination with oxidative polymerization was developed by Wolski et al. [57] to produce conjugated ladder-like polythiophene-based polymer brushes. In this two-step process, 3-methylthienyl methacrylate was polymerized via SI-ATRP giving grafted poly(3-methylthienylmethacrylate) (PMTM) brushes with pendant polymerizable thiophene groups, which after subsequent template polymerization resulted in the formation of ladder-like architecture. Ghosh et al. [58] have synthesized water soluble polythiophene–*g*–poly-[*N*-(6-methyluracilyl)-*N,N*-dimethylaminochloride]ethylmethacrylate (PTDU), which shows thermo-responsiveness in presence of halides and exhibits light induced conformational change. The thermoresponsive behavior was introduced to polythiophene backbone via ATRP of 2-(dimethylamino)-ethyl methacrylate (DMAEMA) from polythiophene macroinitiator (PTI) giving PTDU. Then, cationic PTDU was prepared by the quaternization of amine groups of DMAEMA with 6-chloromethyl uracil (Figure 5). Attachment of the uracil moieties makes the system responsive to diffused light by changing the conformation from extended to coiled structure.

**Scheme 4:**
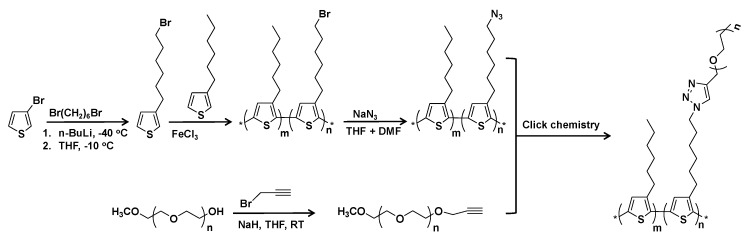
Synthesis of P3HT–*g*–PEO random copolymers through oxidative polymerization and click reactions.

In another interesting example, Strover et al. [59] grafted hydrophilic poly(2-hydroxyethyl methacrylate) on bromo-initiator functionalized conducting polymer poly(2-(2,5-di(pyrrol-2-yl) thiophen-3-yl) (PPyThon) using electrochemically controlled ATRP (eATRP). For the first time this group introduced eATRP as a grafting method where PPyThon was used as a working electrode and macroinitiator as well, from which electrografting of (2-hydroxyethyl methacrylate) occurs giving the graft polymer via ATRP. Recently, Chan et al. [60] prepared thermoresponsive water soluble laterally-branched phenylene derivative of polythiophene (PThP) to develop soluble thermometer. At first, PThP attached with ATRP initiating sites and azide groups in the side chains was prepared and then subjected to ATRP to graft poly(ethylene glycol) methacrylate (PEGMA) generating water soluble conducting polymers. To introduce thermoresponsive behavior, propargyl functionalised poly(2-n-propyl-2-oxazoline) was then grafted via click reaction. Voorhaar et al. [61] prepared self-healing graft copolymer for stretchable and wearable electronics. To introduce self-healing properties, PThP was grafted by poly(acetamidoalkyl acrylate) side chains via controlled radical polymerizations of 3-acetamidopropyl acrylate and 6-acetamidohexyl acrylate. In another example, Mohamed et al. [62] used a combination of oxidative radical polymerization and click reactions to synthesize amphiphilic polythiophene–*graft*–poly(ethylene oxide)(P3HT–*g*–PEO) copolymer. To synthesize this copolymer, random copolymer of 3-hexylthiophene (P3HT) and 3-(6-bromohexylthiophene) (P3HT-Br) were prepared via oxidative polymerization and treated with sodium azide (NaN_3_) to form azido-functionalized polythiophene. Finally, polythiophene–*graft*–poly(ethylene oxide) was synthesized from alkyne terminated PEO via alkyne-azide click reaction (Scheme 4).

**Figure 6:**
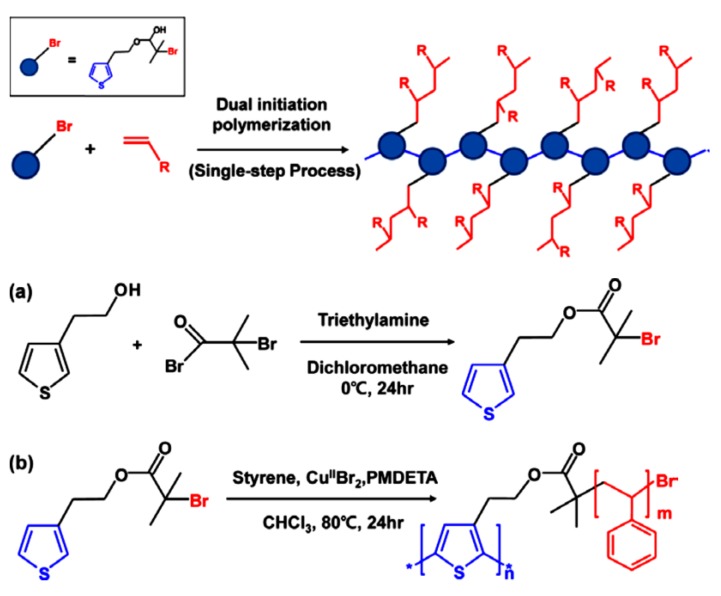
Schematic illustration and synthetic route of the synthesis of *π*-conjugated polymers bearing branched chains (**a**) via the dual initiation polymerization process. (**b**) Reproduced with permission [65].

To explore the properties of all-conjugated graft architectures, Obhi et al. [63] synthesized a comb polymers polythiophene–*graft*–polyselenophene, having polythiophene backbone and polyselenophene side chains. This synthesis was not very straight forward and was done only for low and medium graft density by synthesizing azide-functionalized polythiophene and acetylene-terminated polyselenophene via Kumada catalyst-transfer polycondensation polymerization (KCTP), followed by alkyne-azide click reaction between them. Using Kumada cross-coupling reaction thiophene-functionalized poly(vinyl chloride) (PVC) macromonomer (ThPVCM) was prepared by Massoumi et al. [64] for chemical and electrochemical grafting of polythiophene on PVC. The graft polymer was then synthesized chemically from the macromonomer via oxidative polymerization of thiophene and electrochemically by electrolysis under constant potential. In another interesting study, Hong et al. [65] reported a simple approach to prepare polythiophene containing branched chains in a single step via dual initiation polymerization technique. This approach consists of both oxidative and metal-catalyzed radical polymerization simultaneously in presence of Cu^2+^/Cu^+^ catalyst system that allows polymerizations of both the backbone polythiophene and branched polymers in a single step (Figure 6). 

Apart from the grafting of regular polymers people also use covalent grafting of polypeptide as an alternative approach to composite formation in order to prevent leaching of polypeptides from the scaffold materials. As for example, Akbulut et al. [66] prepared polythiophne–*graft*–polyalanine (PTh–*g*–PAla), which after attachment with glucose oxidase (GOx) enzyme can act as a glucose sensor. For the synthesis, thiophene-functionalized polypeptide macromonomer by the *N*-carboxyanhydride (NCA) ROP was prepared first and then electropolymerization of the macromonomer was done. This biosensor was reported to possess better sensitivity and repeatability due to inhibition of leaching. Moreover, Guler et al. [67] synthesized polythiophene–*g*–polyphenylalanine (PTh–*g*–PPhe) and attached GO*x* to develop similar type of biosensor. Further, RGD was attached to the polyphenylalanine chains of this graft copolymer to promote better cell adhesion.

**Figure 7:**
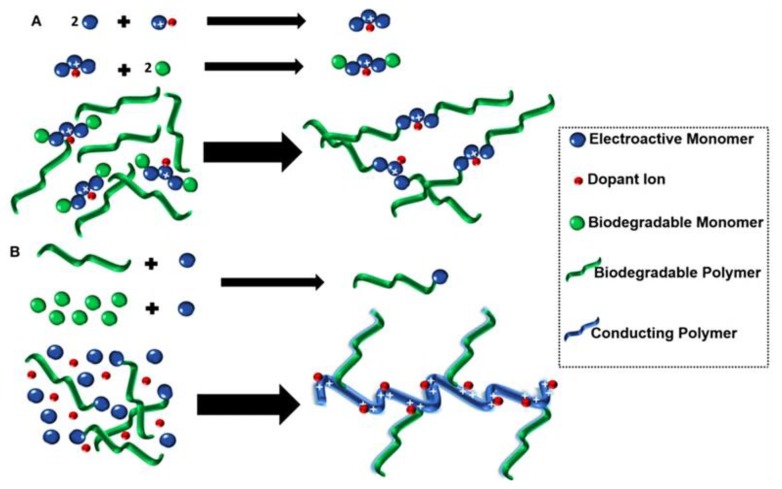
Schematic illustrations for synthesis of (**A**) electroactive oligomers and block copolymers and (**B**) electroactive macromonomers and graft copolymers. Reproduced with permission [68].

In order to develop electroactive biomaterials for biomedical applications, grafting of biocompatible and biodegradable polymers such as poly(caprolactone) (PCL), poly(d,l-lactic acid) (PDLA, PLLA), poly(glycolic acid) (PGA) with conducting polymers has gained much attention as a novel approach. To summarize the developing research on the synthesis of conducting polymer-based biomaterials and improvement of their properties after grafting, very recently, Sliva et al. [68] presented a good review article. Mainly, there are two ways to generate such conducting graft polymers: (i) attachment of electroactive oligomers to the biodegradable polymers via ester linkage (Figure 7A) and (ii) subsequent synthesis of electroactive, biodegradable polyesters macromonomer followed by polymerization of conductive monomers (Figure 7B).

Using the oligomer attachment approach, Chen et al. [69] synthesized a series of biodegradable electroactive shape memory-based polymer network comprising of aniline trimer as hard block, PEG and PLLA as soft blocks for soft tissue engineering applications. By this combination the authors were able to achieve super stretchability and low modulus required to mimic soft tissues. For muscle tissue engineering, the same group developed biocompatible, biodegradable, electroactive copolymers of polyurethane-urea (PUU), PLLA and aniline trimer [70]. The elastomeric properties of PUU combined with the conductive behavior of aniline trimer and biocompatibility, biodegradability of PLLA make it a potential candidate for repair and regeneration of soft tissues like skeletal muscle, cardiac and nerve muscle. Although, oligoaniline (trimer, tetramer, pentamer) is a better substitute of PANI itself in terms of biocompatibility, solubility and processability, but both the materials have harmful effect on cells for long-term in vivo studies. To address this point, Spicer et al. [71] developed a series of oligomers of EDOT for tissue engineering purposes.

Apart from the oligomer approach macromonomer approach is also very effective for the development of degradable conducting graft copolymers. To exemplify, PCL grafted PPy was synthesized by Guo et al. [72] aiming the fibrous membrane formation for biomedical applications. First, pyrrole–*g*–PCL (Py–*g*–PCL) macromonomer was synthesized using ROP of caprolactone. Then, PPy–*g*–PCL was achieved by oxidative polymerization of Py–*g*–PCL monomer. Recently, Sliva et al. [73] reported a series of copolymer of PEDOT with poly(d,l-lactic acid) (PEDOT–*co*–PDLLA) as a scaffold materials for neuronal tissue engineering. The copolymers were synthesized via subsequent EDOT−PDLLA macromonomer production using ROP of lactide in presence of EDOT-OH, followed by chemical copolymerization of EDOT monomers.

**Table 1:** Representative important research works done in the field of covalent grafting of conducting polymers.

Conducting Polymer System	Grafted/Attached System	Approach	Outcome	Ref.
poly(3,4-ethylenedioxythiophene)	Poly(acrylic acid)	ATRP, followed by acid hydrolysis of tert-butyl acrylate	Functional biointerface and versatile cell culture substrate.	[37]
poly(o-methoxyaniline)	Polyacrylamide	Oxidative-radical coupling	Wastewater treatment	[44]
Polyaniline	Xanthan gum	Oxidative polymerization	Room temperature ammonia vapor sensor	[45]
Polyaniline	Novolac	Oxidative polymerization	Conductive adhesive	[47]
Polyaniline	Polyacrylamide	Electrospinning of copolymers in a mixed solvent	Potential electroconductive fibrous mat for supercapacitors	[48]
Polypyrrole	Poly(Schiff base) and poly(ethylene glycol)	Electrochemical co-polymerization of pyrrole and pre-synthesized macromonomer.	Amphiphilic conducting graft copolymer based implantable electrode for serotonin detection	[50]
Aniline tetramer	Dextran and (4-formylbenzoic acid).	Step-wise grafting of dextran and 4-formylbenzoic acid via polycondensation coupling reaction from hexamethylene diisocyanate-graft-aniline tetramer	Degradable, conductive, self-healing injectable hydrogel for myoblast cell therapy and muscle repair	[52]
Polythiophene	Poly(3-methylthienylmethacrylate)	Self-templating SI-ATRP combined with oxidative polymerization	Formation of conjugated ladder-like architecture	[57]
Polythiophene	poly-[*N*-(6-methyluracilyl)-*N,N*-dimethylaminochloride]ethylmethacrylate	ATRP followed by quaternization of amine groups	Water soluble conducting brush exhibiting light-Induced conformational change and thermo-responsiveness in presence of halides.	[58]
Poly(2-(2,5-di(pyrrol-2-yl) thiophen-3-yl)	Poly(2-hydroxyethyl methacrylate)	Electrochemically controlled ATRP (eATRP)	New grafting method to produce hydrophilic conducting graft copolymers.	[59]
Phenylene derivative of polythiophene	Poly(ethylene glycol) methacrylate and propargyl functionalised poly(2-n-propyl-2-oxazoline)	ATRP followed by click reaction	Soluble thermometer	[60]
Phenylene derivative of polythiophene	Poly(acetamidoalkyl acrylate)	ATRP	Self-healing, stretchable and wearable electronics	[61]
Polythiophene	Poly(ethylene oxide)	Combination of oxidative radical polymerization and click reactions	More processable amphiphilic conducting system.	[62]
Polythiophene	Polyselenophene	KCTP followed by click reaction	Production of all-conjugated comb-like graft architectures	[63]
Polythiophene	Polystyrene	Single step dual initiation polymerization technique consisting of both oxidative and metal-catalyzed radical polymerization simultaneously	Simple single step approach to prepare conducting polymer containing branched chains.	[65]
Polythiophene	Polyalanine	First ROP, then electropolymerization	Glucose sensor	[66]
Aniline trimer	Poly(ethylene glycol) and poly(l-lactic acid)	Coupling via polycondensation reaction.	Biodegradable shape memory-based superstretchable electroactive elastomer network for soft tissue engineering.	[69]
Polypyrrole	Poly(caprolacton)	First ROP, then oxidative polymerization	Degradable, fibrous, conducting scaffold materials for neuronal tissue engineering	[72]

**Figure 8:**
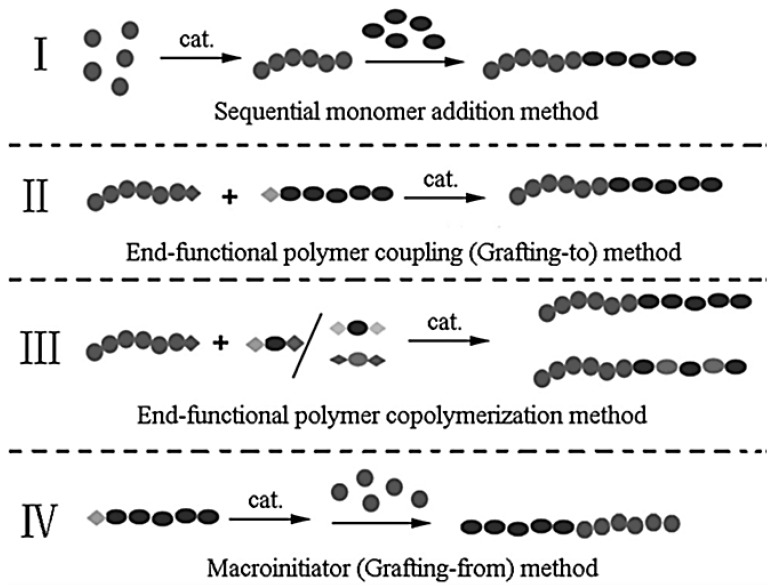
Synthetic approaches of all-conjugated D–A block copolymers. (The lighter shaded balls stand for p-type monomers or p-type polymers, and the darker one stands for n-type monomers or n-type polymers). Reproduced with permission [74].

In recent years, the development of all-conjugated donor-acceptor based polymer solar cell has gained high attention because the presence of both donor and acceptor segments in a single polymer chain can contribute easily to fabricate high performance solar cell. The general methods for the synthesis of all-conjugated donor-acceptor graft copolymers are beautifully summarized in a mini-review article by Wang et al. [74]. The synthetic routes (Figure 8) are categorized into four types: (i) step-wise monomer addition via catalyst transfer polycondensation, (ii) attachment of end-terminal P3HT and end-functionalized n-type polymers (iii) copolymerization of end-functionalized conducting polymers (mainly P3HT) with n-type monomers by Suzuki coupling, Yamamoto coupling, Stille polycondensations, etc. and (iv) catalyst-transfer polycondensation from acceptor polymers based macroinitiator. 

The above examples give a good idea about how the covalent grafting of conducting polymers are a steadily growing area. The key issue in this effort is to define the purpose of grafting. The nature of grafted materials entirely depends on that purpose. Therefore, the same conducting polymer backbones are being grafted with various polymeric or oligomeric materials. In the covalent grafting approach, the grafted materials have a high degree of contribution to the overall system. Therefore, complementarity in property and function makes this approach unique.

## 3. Non-Covalent Grafting of Conducting Polymers

The term grafting more commonly refers to a covalent attachment to the polymer backbone. However, in cases, covalent grafting might have its own demerit too. Complex synthetic design and modification techniques, undesired property modification, irreversible nature of attachments, are a few key issues which prompted chemists to think about a more adaptive approach. Such concerns give birth to an entirely unconventional grafting strategy where the associated components stay together via noncovalent bonding. Therefore, one might also term this as supramolecular grafting.

To date, there are only a few reports on supramolecular grafting with nonconducting polymer, which for example, utilized quadruple hydrogen bond formation [75], and host-guest complexation between adamantyl group and β-cyclodextrin [76], or between ferrocene and β-cyclodextrin [77]. The importance and usefulness of supramolecular grafting approach is perhaps even more important for conducting polymers compared to the nonconducting polymer family. While grafting of the conducting polymer can introduce the desired durability and processability on top of its unbeatable electrical and electronic properties, in cases, direct attachment to the conducting polymer backbone might have detrimental effect on the main chain conjugation. Therefore, supramolecular attachment to the conducting polymer might add the property of the grafted species and also minimize the main chain perturbation at the same time. As this particular area is not fully developed and there are only limited reports available in the literature, interesting works involving polymer–polymer supramolecular attachments will also be included in the present discussion as the pathway toward efficient and successful supramolecular grafting. Selected works on non-covalent grafting of conducting polymers are summarized in Table 2.

**Scheme 5:**
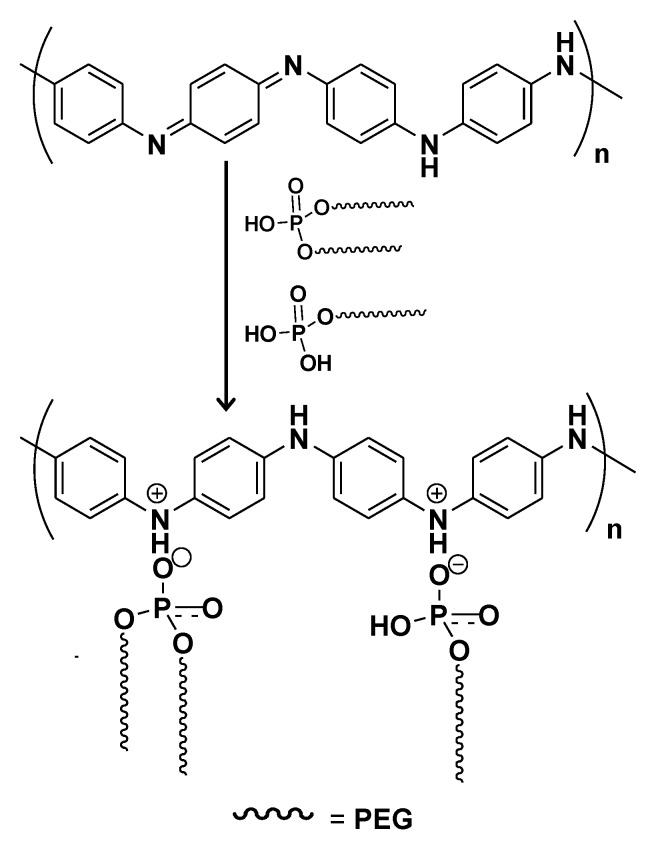
Structure of the PEG derived phosphoric acid doped PANI.

**Figure 9:**
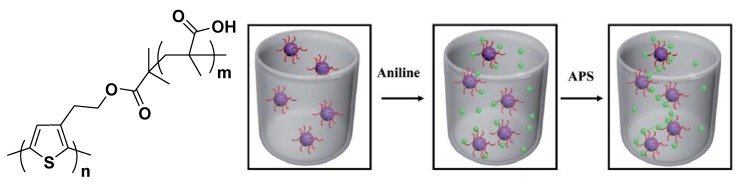
Chemical structure of grafted polythiophene dopant (**left**) and schematic illustration of hybrid helical structure formation. Violet spheres stand for polythiophene core, red hairs correspond to polymethacrylic acid chain, green spheres stand for aniline molecule and green chain with green spheres represents polyaniline. Reproduced with permission [80].

In order to prepare soluble polyaniline, in one of the earliest examples, Geng et al. doped polyaniline (PANI) by phosphonic acid containing hydrophilic tail. When poly(ethylene glycol) monomethyl ether (PEGME)with molecular weight *M*_w_ 550 was used as the hydrophilic chain of the dopant, the doped polymer was soluble in water, NMP and chloroform [78]. Free-standing films could also be cast from water, chloroform and NMP solutions. The film cast from aqueous solution showed good electrochemical redox reversibility. Interestingly, reversible phase transformations in DSC spectra at 19.1 and −9.8 °C for heating and cooling curve respectively, was observed due to the melting and crystallization events of hydrophilic PEGME segments. In this system design, the attachment of two polymeric components are ionic in nature. Later, self-assembly property of the same composite system was studied by Nandan et al., where the strong PANI backbone-PEG side chain repulsion resulted in a microphase-separated lamellar morphology consisting of alternating ionic layer composed of PANI backbones and ionic headgroups of the dopant and nonionic layers consisting of PEG side chain (Scheme 5) [79]. The thickness of the nonionic layer increased with increasing binding fraction because of higher chain stretching as the separation distance between the junction points at the PANI backbone increased. In addition, the lamellar interface was found to be almost planar. An order–disorder transition near 225 °C due to deprotonation in the complex was captured from temperature dependent SAXS studies. The conformational rigidity of PANI backbone coupled with the strong backbone-side chain ionic attachment strongly retarded the crystallization kinetics and the crystallizability of the PEG side chains in the complexes. In general, conformationally flexible PEG segment experienced a higher degree of property modification compared to the conformationally rigid PANI backbone. Another example of a doping approach for attaching polymeric component to PANI backbone is the work by Nandi and co-worker, where, interestingly, the attached polymer itself was another conducting polymer [80]. In this work, polythiophene–*g*–poly(methacrylic acid) (PTMA) was prepared by ATRP of tert-butyl methacrylate upon a polythiophene backbone followed by hydrolysis of tert-butyl groups. The resultant grafted polythiophene acted as both a template and a dopant for the synthesis of PANI nanostructures by oxidative polymerization of aniline. A nanorod hybrid morphology was observed up to a 1:10 composition (PTMA–PANI; *w*/*w*). On the other hand, for 1:20 composition a helical nanorod was produced along with some small size spheroids. The hybrids were semiconducting in nature and exhibited reproducible photoconductivity by alternate “On” and “Off” switching of white light. Polymerization of adsorbed aniline on the surface of the micellar polythiophene moiety has been postulated and considered responsible for helical PANI chain and variable morphology based on the relative ratios of involved conducting polymer components (Figure 9).

**Figure 10:**
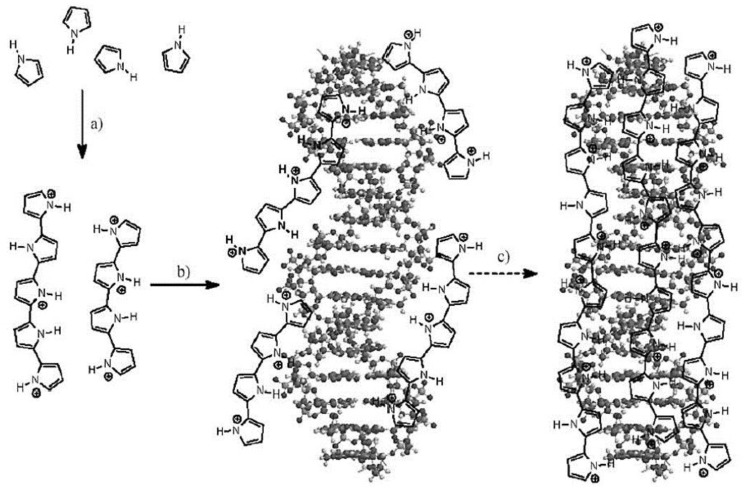
Proposed mechanism of self-assembly of DNA/PPy nanowires. (**a**) Oxidation of pyrrole monomer with FeCl_3_, (**b**) association of oligomers on DNA through supramolecular interactions, (**c**) polymer growth on DNA template. Reproduced with permission [85].

**Figure 11:**
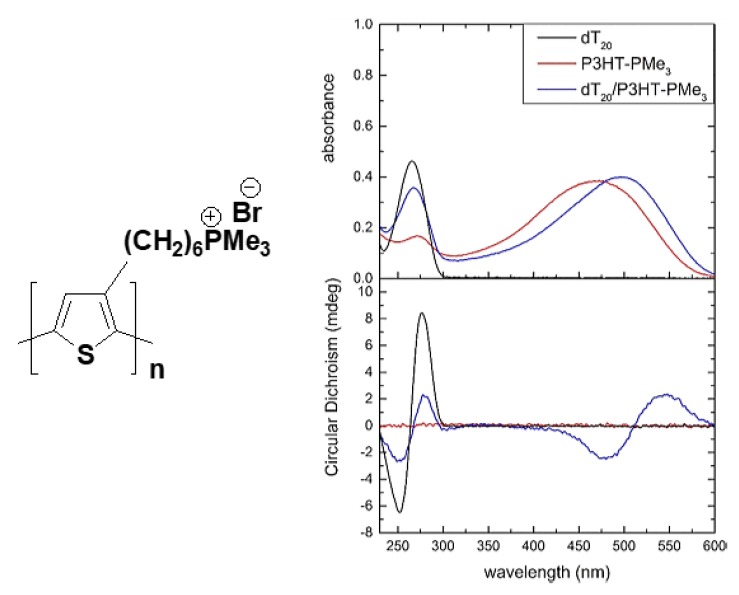
Chemical structure of the cationic (P3HT-PMe3) (**left**) and UV-Vis and CD spectra of dT20 (black line), P3HT-PMe3 (red line) and dT20(oligonucletide)/P3HT-PMe3 complex (blue line) at a 1:1 DNA/polymer charge ratio. Reproduced with permission [86].

A similar approach involving supramolecular polar interaction could be adopted to form other conducting polymer hybrids. Electrostatic interaction between cationic conducting polymer and anionic biopolymer like DNA has been used successfully to align two polymeric materials. The key difference with conventional grafted system is that here the attachment is multipoint along the parent polymer backbone. A supramolecular hybrid formation between poly(o-methoxyaniline) (POMA) and DNA was utilized by this author as a tool for the formation of semiconducting DNA hybrid and POMA radical cation stabilization. An uncoiling of POMA chain on the DNA template facilitated by the attractive interaction between DNA anion and POMA radical cation and repulsive interaction among POMA radical cations was key event involved in this stable hybrid formation [81,82,83]. Such a system was further utilized to produce a large-band-gap semiconducting nano-biocomposite in presence of silver ion [84]. Similarly, DNA/polypyrrole supramolecular hybrid by oxidation of pyrrole in DNA-containing solution was achieved by Dong et al. [85]. Interestingly, reactions carried out on surface-immobilized DNA and on DNA free in solution resulted different nanostructures. The former method resulted in a beads-on-a-string appearance for the strands, whereas, nanowires prepared in solution had continuous coverage of DNA (Figure 10). The latter type of strand was electrically conducting and conformationally flexible, allowing alignment of the polymer nanowires by molecular combing, which can be considered a convenient way of fabricating a simple electrical device by stretching DNA/PPystrands across an electrode gap. Expanding this approach to other conducting polymer, supramolecular assembly of poly[3-(6’-(trimethylphosphonium)hexyl)thiophene-2,5-diyl] (P3HT-PMe3) with single-stranded oligonucleotides or long genomic DNA has been studied in a very recent work by Leclercq et al. [86]. Self-assembly of two polymeric components in buffered aqueous solution yielded polyplexes with hydrodynamic radii ranging from 7 nm to around 25 nm. In these polyplexes, chirality was transcribed to achiral polymer from chiral DNA in supramolecular manner as evidenced from circular dichroism spectra (Figure 11). When the hybrid solution is deposited on a surface formation of dendritic fibers extending over tens of μm was observed. Such chemistry has prospective applications in cell transfection, or self-assembled fibers for organic bioelectronics.

**Figure 12:**
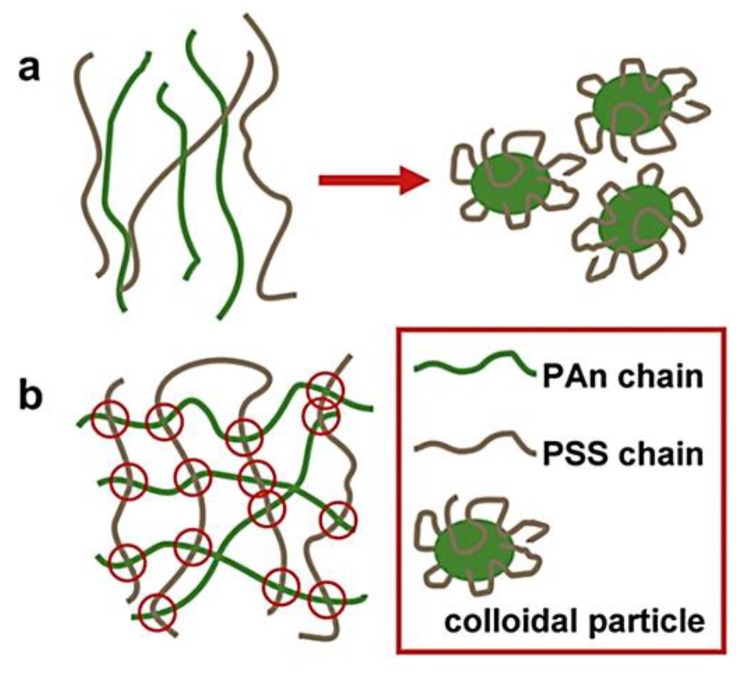
Illustration showing the effects of the reagent concentrations on the formation of the molecular networks. (**a**) In dilute solutions, PANI and PSS chains spontaneously assemble to colloidal particles. (**b**) In concentrated solutions, molecular network structures are formed and established. The red circles denote the linking points of the networks, where the interactions between the polymer chains take functions. Reproduced with permission [87].

Utilizing a similar electrostatic protocol, polyaniline-poly(styrene sulfonate) (PANI-PSS) hydrogels have been developed by Dai et al. [87]. A hierarchical porous microstructure consisting of oriented 1D nanofibers was observed in the hydrogels (Figure 12). The hydrogels transformed into colloidal particles in alkaline solutions because of dedoping of PANI. Interestingly, the PANI-PSS hydrogels demonstrated improved capacitance performance such as higher energy density, higher power density and better electrochemical stability, compared to the conventional PANI-PSS colloids.

**Figure 13:**
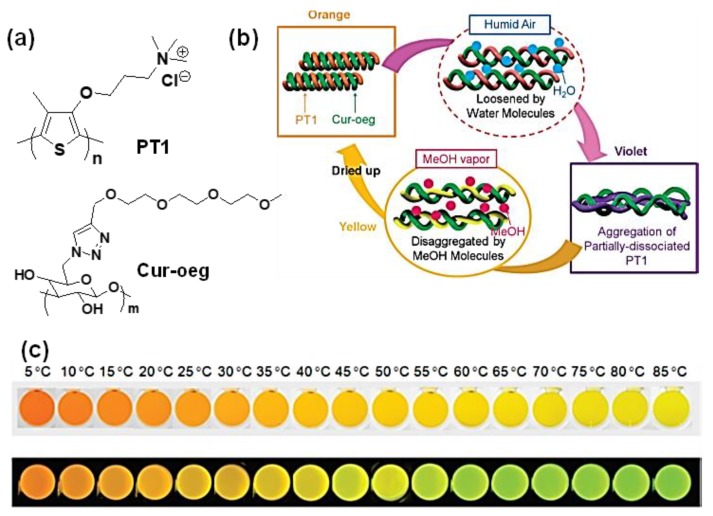
(**a**) Chemical structure of the polymeric components, (**b**) schematic representation of the vapor-induced color change in PT1/Cur-oeg complex film and (**c**) photographs of the PT1/Cur-oeg complex solution under the temperature control from 5 to 85 °C; upper line: bright images, lower line: fluorescence images (*λ*_ex_ = 365 nm), [PT1]_unit_ = 0.2 mM, [Cur-oeg]_unit_ = 0.6 mM, in water containing 5 vol % DMSO. Reproduced with permission [89].

In addition to polar interaction, solvophobic interaction can be a leading driving force for supramolecular association. Shinkai and co-workers pioneered in using β-1,3-Glucan polysaccharides as novel one-dimensional hosts for various species including conducting polymers [88]. In one of their works, they used a modified polysaccharide (Curoeg) to form a “loose” but helical macromolecular complex with an achiral cationic polythiophene (PT1) [89]. The effective conjugation length of PT1 was changed consistently with temperature between 5 and 85 °C (Figure 13). As the color changes in the absorption and the fluorescence were detectable by naked eye and are reversibly controlled under thermal cycles, authors termed this system as molecular thermometer. Interestingly, the polymeric complex also showed vapor chromism in the film state (Figure 13). In solution as well as in film state the driving factor was the conformational rearrangement of the conducting polymer on the polysaccharide surface in presence of stimuli. The significant part of this study is that because of the nonpolar type of interaction conducting polymer backbone or its electronic property was least influenced because of the attachment.

**Figure 14:**
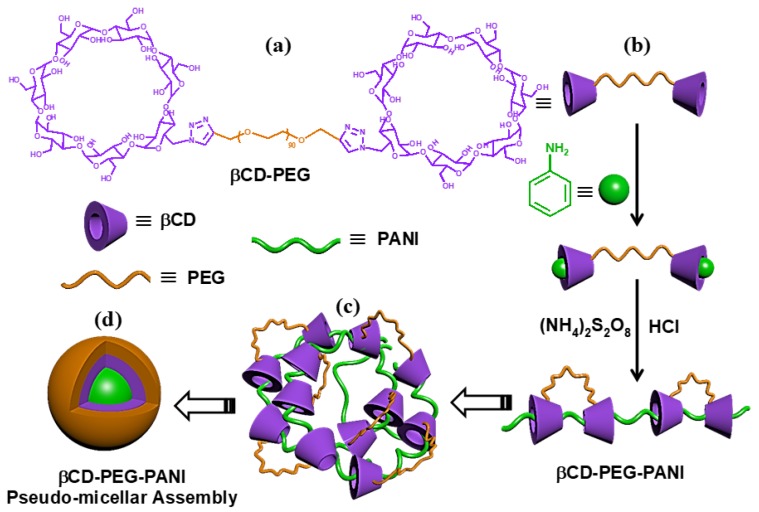
(**a**) Molecular structure of βCD-PEG, (**b**–**d**) simplified illustration of formation and assembly of βCD-PEG-PANI. Here, the involvement of βCD units (attached to PEG chains), during polymerization is considered random. Reproduced with permission from [90].

As mentioned earlier, the hypothesized effectiveness of supramolecular grafting lies in its minimal interference with the electronic and electrical properties of conducting polymer. Therefore, a host-guest interaction mediated grafting strategy could be promising technique because of two key reasons: first, nonpolar nature of attachment will ensure retention of electronic property along the conducting polymer backbone; and second, strong host-guest binding could reproduce the close association of covalent grafting for significant property mixing. To the best of our knowledge, the only work of supramolecular conducting polymer grafting in its true sense is reported by these authors, where PANI was grafted with PEG coupled with b-cyclodextrin (βCD-PEG) forming a pseudorotaxane with the aniline moiety [90]. The supramolecularly grafted PANI (βCD-PEG-PANI) in the doped state showed an extremely high solubility in aqueous as well as in organic solvents. Interestingly, the grafted PANI exhibited a higher degree of doping and a highly efficient radical cation stabilization, compared to a control PANI system synthesized under identical conditions. It is worth noticing that the redox switching property of PANI was fully retained in the grafted state. In addition, a novel disk-like morphology was observed in the βCD-PEG-PANI system. A pseudo-micellar assembly formation of βCD-PEG-PANI could explain the high solubility, efficient radical cation stabilization, and morphology of the grafted PANI (Figure 14). The restricted conformation in a pseudo-micellar assembly structure in solution, forming a uniform disk-like morphology was the cause behind efficient radical cation stabilization and a lesser electron delocalization in βCD-PEG-PANI. The present system represents a powerful integration of three key elements: first, fascinating electronic and physicochemical properties of parent PANI; second, extremely high aqueous solubility; and third, biocompatibility transcribed from PEG and βCD. The self-assembled supramolecularly grafted system exhibited adaptivity towards PANI-DNA complexation in aqueous solution, which could be considered a step forward towards biocommunication.

**Table 2:** Representative important research works done in the field of non-covalent grafting of conducting polymers.

Conducting Polymer System	Grafted/Attached System	Approach	Outcome	Ref.
Polyaniline	Poly(ethylene glycol)	Acid doping: Electrostatic attachment	Solubility enhancement, film formation	[78,79]
Polythiophene	Poly(methacrylic acid)	ATRP	Novel hybrid morphology; Electrical and photoconduction	[80]
Poly(o-methoxyaniline)	DNA	Acid doping: Electrostatic attachment	Enhanced radical cation stabilization; Semiconducting hybrid	[81,82,83]
Poly(o-methoxyaniline)	DNA and silver	Redox and electrostatic	Large-band-gap semiconductor	[84]
Polypyrrole	DNA	In-situ polymerization in DNA solution	Distinct nanostraucture based on surface or bulk polymerization	[85]
Polythiophene derivative	Oligonucleotide, DNA	Co-assembly in buffer	Formation of extended dendritic fiber	[86]
Polyaniline	Poly(styrene sulfonate)	Doping: electrostatic attachment	Porous microstructure; Improved capacitance performance	[87]
Cationic polythiophene	Polysaccharide	Electrostatic	Hybrids with temperature responsiveness in solution and vapor responsiveness in film state	[89]
Polyaniline	Poly(ethylene glycol)	Pseudo rotaxane formation	Solubility enhancement; morphology transition; high radical cation stability; adaptive complexation with DNA in aqueous medium	[90]

Authors believe that in supramolecular grafting the synchronization of additive approach and adaptive behavior can revolutionize polymer modification techniques to address multiple issues associated with processability and device fabrication based on conducting polymers. Finally, the non-covalent mode of attachment of the monomer implies that a strategy applied to one conducting polymer can potentially be employed for a wide range of conducting polymers.

## 4. Outlook

Grafting is undoubtedly a fascinating expansion of the conducting polymer family bridging its chemical assets with the real-world demands. Unparallel electronic and electrical property together with environmental responsiveness can couple sensibly with flexibility, processability and biocompatibility of nonconducting traditional polymeric systems to generate many breakthroughs in technology generation and biocommunication strategy. Apart from conducting and non-conducting polymer pair, association or co-assembly of multiple conducting polymers in the same system could also be an excellent extension of the above chemistry.
